# Time-Dependent Risk of Psychiatric Disorders in Pediatric and Adolescent Patients with Microtia: A Nationwide Population-Based Cohort Study

**DOI:** 10.3390/jcm15082998

**Published:** 2026-04-15

**Authors:** Jeong Yeop Ryu, Seok Gi Kim, Joon Seok Lee, Jung Dug Yang, Ho Yun Chung, Kang Young Choi

**Affiliations:** 1 Department of Plastic and Reconstructive Surgery, School of Medicine, Kyungpook National University, Daegu 41405, Republic of Korea; seokgi1004@naver.com (S.G.K.); leejspo2025@knu.ac.kr (J.S.L.); lambyang@knu.ac.kr (J.D.Y.); chunghoyun@gmail.com (H.Y.C.); 2 Cell and Matrix Research Institute, School of Medicine, Kyungpook National University, Daegu 41405, Republic of Korea

**Keywords:** congenital microtia, mental disorders, attention deficit disorder with hyperactivity, autistic disorder

## Abstract

**Background/Objectives**: Microtia, a congenital ear malformation ranging from mild anomalies to anotia, imposes a psychosocial burden, yet its link to pediatric psychiatric disorders in population-based settings is understudied. **Methods**: This study included 6048 patients with microtia and a control cohort of 120,960 age- and sex-matched participants from the Korean National Health Insurance database. The incidence of overall psychiatric disorder events was compared between the microtia and control cohorts. **Results**: Compared with the control cohort, the incidence rate ratio (IRR) in patients with microtia was 1.16 (95% CI, 1.05–1.29) for overall psychiatric disorders, 5.35 (95% CI, 4.56–6.27) for hyperkinetic disorder (HKD), and 9.67 (95% CI, 5.49–17.03) for autism. After adjusting for age group, sex, and socioeconomic status, microtia was associated with an increased risk of overall psychiatric disorders, HKD, and autism. Subgroup analyses revealed an elevated risk of overall psychiatric disorders among individuals aged 5 to 14 years, males, and those with low or high socioeconomic status. Ear reconstruction surgery was associated with a lower risk of HKD among patients aged 10 to 14 years, females, and those with low socioeconomic status. The observed association between ear reconstruction surgery and lower HKD risk in these specific subgroups warrants further investigation using study designs capable of establishing causal inference. **Conclusions**: Microtia is associated with an increased risk of psychiatric disorders in pediatric and adolescent populations, with particularly pronounced risks for HKD and childhood autism. These findings underscore the importance of early psychiatric screening in children with microtia.

## 1. Introduction

Microtia is a congenital malformation of the external ear, characterized by underdevelopment or absence of the auricle, ranging from mild structural anomalies to complete anotia. The prevalence of microtia varies significantly, ranging from 0.83 to 17.4 per 10,000 births, with elevated rates in Hispanic, Asian, and Native American populations. Microtia predominantly affects males and typically presents unilaterally, often on the right side [1,2,3,4]. It may occur as an isolated condition or in association with craniofacial syndromes (e.g., hemifacial microsomia and Treacher Collins syndrome) and is frequently accompanied by conductive hearing loss due to external auditory canal atresia [3,4,5].

The visible nature of microtia can lead to significant psychosocial challenges, particularly in pediatric populations. Affected children may experience social stigma, peer teasing, and reduced self-esteem, which are well-documented risk factors for psychiatric distress. Previous studies have reported elevated rates of psychiatric issues among patients with microtia, including depression (20.2%), interpersonal sensitivity or social difficulties (36.6%), and hostility or aggression (26.3%) [6]. These findings suggest that microtia may contribute to psychiatric vulnerabilities, potentially amplified by environmental factors such as family dynamics, parenting styles, or socioeconomic challenges. Additionally, conductive hearing loss associated with microtia may impair speech and language development, further increasing the risk of attention deficit disorders and social isolation.

Despite the recognized psychosocial burden of microtia, its association with pediatric psychiatric disorders in population-based settings remains underexplored. Unlike studies focusing on clinical cohorts, population-based approaches offer a broader perspective on the prevalence and risk factors of psychiatric outcomes, capturing a more representative sample of affected individuals. This nationwide population-based study examines the association between microtia and pediatric psychiatric disorders to provide evidence guiding clinical management and preventive strategies for affected children. It compares these children with age- and sex-matched controls using the Korean National Health Insurance (NHI) claims database.

## 2. Materials and Methods

### 2.1. Ethical Approval and Data Source

This study was approved by the Institutional Review Board of Kyungpook National University Hospital (IRB No. KNUH 2023-11-030) and performed in accordance with the principles of the Declaration of Helsinki. All personal information was anonymized.

The NHI database in South Korea encompasses more than 97% of the population and contains comprehensive details on medical practices throughout the country. It includes personal data such as age, sex, and regional information of enrollees; socioeconomic details, including income levels; complete records of outpatient and inpatient claims; and diagnostic and treatment codes based on the International Classification of Diseases, Tenth Revision (ICD-10) [7,8,9,10,11,12,13,14]. Additionally, the South Korean government operates the National Health Insurance Service-Infants and Children’s Health Screening (NHIS-INCHS) program, which ensures that all infants and children born in South Korea undergo examinations by pediatricians and dentists. These records are stored in the NHI claims database and are accessible upon request [8].

### 2.2. Study Population

**Figure 1:**
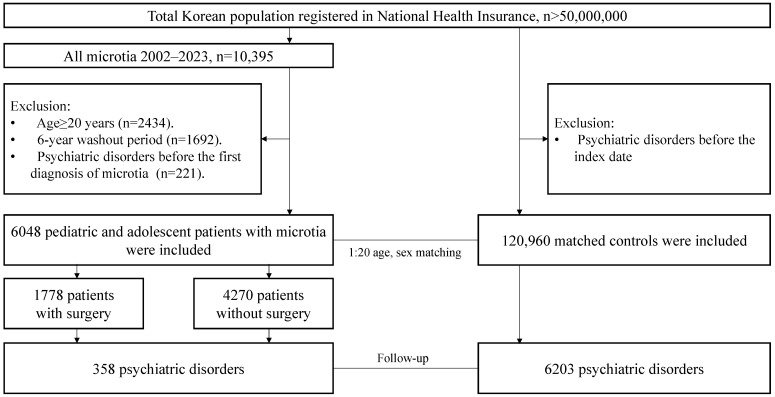
Flowchart of the study.

Data of patients with microtia diagnosed between January 2008 and December 2023 were extracted from the NHI database (Figure 1). Microtia was defined as having visited the clinic more than two times with the ICD-10 code Q16.0 or Q17.2. A six-year washout period was applied to define newly diagnosed patients with microtia, between January 2002 and the timing of diagnosis of microtia (the “index date”). This washout duration was selected because the NHIS database provides complete claims data from 2002 onward, ensuring adequate exclusion of prevalent cases. To limit our study to patients diagnosed with microtia during childhood or adolescence (aged 0–19 years), we excluded those aged 20 years or older. Patients diagnosed with psychiatric disorders before the diagnosis of microtia were also excluded. Among patients with microtia, ear reconstruction surgery was defined as procedures including ear reconstruction with autologous rib cartilage (N054X), ear lobule rotation (SB161), a skin flap or temporoparietal fascia flap (SB167), a full-thickness skin graft on the face (S0171), and a split-thickness skin graft (N0173).

The control cohort was also extracted from the NHI database. For every patient with microtia, 20 microtia-free individuals on the “index date” were sampled from the general population. This cohort was matched by birth year and sex. Controls were selected without replacement.

### 2.3. Socioeconomic Classification

The Korean NHI system calculates premiums based on individual earnings, allowing researchers to evaluate socioeconomic status across the population. Because pediatric and adolescent patients with microtia lack personal income, the NHI designates these minors as dependents, with parents or guardians as subscribers, determining 20 income tiers based on the subscriber’s income. For this study, income data from patients’ parents or guardians were classified into 20 tiers, with the lowest tertile defined as low income, the middle tertile as middle income, and the highest tertile as high income [14]. This income-based classification represents a proxy for socioeconomic status derived from NHIS premium tiers and does not incorporate other dimensions such as educational attainment, occupation, or household size.

### 2.4. Study Outcomes

The primary study outcome was the initial diagnosis of a psychiatric disorder, specifically including depressive disorder, anxiety disorder, stress-related and adjustment disorders, conduct disorder, hyperkinetic disorder (HKD), childhood autism, Asperger syndrome, and dysthymia. These were defined by ICD-10 code-based diagnoses and corresponding psychotherapy administration. ICD-10 codes were as follows: depressive disorder (F32–F33), anxiety disorder (F40–F41), stress-related and adjustment disorders (F43), conduct disorder (F91), HKD (F90), childhood autism (F84.0), Asperger syndrome (F84.5), and dysthymia (F34.1). Treatment modalities for mental disorders were identified using codes for psychotherapies, including individual psychotherapy (NN011–NN013), group psychotherapy (NN021–NN023), and family therapy (NN031–NN032) [15]. A psychiatric disorder event was defined as the first claim record containing both the relevant ICD-10 diagnostic code and a psychotherapy procedure code, regardless of whether these occurred at a single visit or across separate visits. This dual-criterion definition was adopted to enhance diagnostic specificity and ensure that identified cases represented clinically significant disorders necessitating active treatment.

### 2.5. Diagnostic Accuracy

To assess diagnostic accuracy, we randomly selected 150 patients aged 0 to 19 years with ICD-10 diagnostic codes Q16.0 or Q17.2 for microtia, who visited the clinic more than twice between 2008 and 2023. The sensitivity and specificity of the diagnostic algorithm were evaluated, revealing a diagnostic sensitivity of 94% and a specificity of 99.33%.

### 2.6. Sensitivity Analysis

A sensitivity analysis was performed to determine whether disparities in healthcare utilization between patients with microtia and controls influenced the study outcomes. Data on the number of outpatient visits and duration of hospital stays were collected for both groups during the year preceding the index date. The median number of outpatient visits was 6 (estimated 95% CI, 6) for both patients with microtia and controls. Similarly, the median length of hospital stay was 1 day (estimated 95% CI, 1) for both cohorts.

### 2.7. Statistical Analysis

This study examined the risk of psychiatric disorders in pediatric and adolescent patients diagnosed with microtia, stratified by age groups, sex, and familial income. Incidence rates (IRs) were defined as the number of psychiatric disorder events per 1000 person-years, and IRs and incidence rate ratios (IRRs) were analyzed by age groups (0–4, 5–9, 10–14, and 15–19 years) and sex. Kaplan–Meier curves illustrated the cumulative incidence of each psychiatric disorder in the microtia and control cohorts. Follow-up began on the index date and was censored at the date of psychiatric disorder diagnosis or 31 December 2023. Additionally, the Cox proportional hazards model was used to assess the independent association between microtia and each psychiatric disorder, with hazard ratios (HRs) and 95% CIs calculated. Multivariate adjustment was performed for age groups, sex, and familial income. For psychiatric disorders where microtia was identified as an independent association, a stratified multivariate Cox analysis was conducted to compare patients with microtia who underwent surgery with those who did not, evaluating the association between surgery and psychiatric disorders. For this surgical subgroup analysis, follow-up for patients who underwent ear reconstruction surgery was initiated on the date of surgery, and only psychiatric events occurring after this date were included as outcomes. For non-surgery patients, each individual was matched to a surgery patient by age and sex, and the follow-up start date was set to the mean surgery date of the matched surgery group. This approach was designed to mitigate immortal time bias by excluding the pre-surgery period from the surgery group’s at-risk time and aligning the temporal starting points of both groups.

All statistical tests used a 2-tailed 95% CI, with significance set at *p* < 0.05. Analyses were conducted using Stata/MP version 19.5 (StataCorp, College Station, TX, USA).

### 2.8. Use of AI-Assisted Tools

During the preparation of this manuscript, an AI-based language model (Claude, Anthropic) was used solely for English language editing and proofreading. The AI tool was not involved in the study design, data collection, statistical analysis, interpretation of results, or generation of scientific content. All AI-assisted edits were critically reviewed and approved by the authors, who assume full responsibility for the final content of this work.

## 3. Results

### 3.1. Characteristics of the Study Population

The study included a total population of 127,008 participants, consisting of a microtia cohort (*n* = 6048) and a control cohort (*n* = 120,960), with the control cohort serving as a 1:20 age- and sex-matched comparison group. Among these patients, male subjects totaled 79,569 (microtia cohort, *n* = 3789; control cohort, *n* = 75,780), and female subjects totaled 47,439 (microtia cohort, *n* = 2259; control cohort, *n* = 45,180). Given the age- and sex-matched design, the age distribution was comparable across cohorts, with 58.96% aged 0 to 4 years, 16.27% aged 5 to 9 years, 15.44% aged 10 to 14 years, and 9.33% aged 15 to 19 years in the total population. Sex distribution showed 62.65% male and 37.35% female participants in both cohorts. Income levels, based on 20 tiers determined by parental or guardian income, were categorized into tertiles. In the total population, the microtia cohort had 16.11% low income, 32.57% middle income, and 48.15% high income, compared with 16.00%, 42.23%, and 39.94% in the control cohort, with 3.17% and 1.83% missing data, respectively. Regarding microtia surgery, 70.6% of the total microtia cohort did not undergo surgery, while 29.4% did. Among male subjects, 69.12% had no surgery and 30.88% had surgery, compared with 73.09% and 26.91% among female subjects, respectively (Table 1).

### 3.2. Incidence Rates of Psychiatric Disorders

The cumulative incidence of psychiatric disorders is shown in Supplementary Figures S1–S9. Incidence rates (IRs) were evaluated in the microtia (358 events over 51,758.23 person-years) and control cohorts (6203 events over 1,043,060.9 person-years). The overall IR for any psychiatric disorder was 6.92 per 1000 person-years (95% CI, 6.24–7.67) in the microtia cohort and 5.95 per 1000 person-years (95% CI, 5.80–6.10) in the control cohort, with an incidence rate ratio (IRR) of 1.16 (95% CI, 1.05–1.29). Specific disorders exhibited varied patterns: depressive disorder IR was 2.09 (95% CI, 1.73–2.52) vs. 3.53 (95% CI, 3.42–3.65) (IRR, 0.59; 95% CI, 0.49–0.71); anxiety disorder, 1.64 (95% CI, 1.33–2.03) vs. 2.36 (95% CI, 2.27–2.45) (IRR, 0.70; 95% CI, 0.56–0.86); stress-related and adjustment disorders, 0.95 (95% CI, 0.72–1.25) vs. 1.15 (95% CI, 1.08–1.21) (IRR, 0.82; 95% CI, 0.62–1.09); conduct disorder, 0.19 (95% CI, 0.10–0.36) vs. 0.11 (95% CI, 0.09–0.13) (IRR, 1.82; 95% CI, 0.98–3.37); HKD, 2.94 (95% CI, 2.51–3.44) vs. 0.55 (95% CI, 0.51–0.60) (IRR, 5.35; 95% CI, 4.56–6.27); autism, 0.23 (95% CI, 0.13–0.41) vs. 0.02 (95% CI, 0.02–0.04) (IRR, 9.67; 95% CI, 5.49–17.03); Asperger syndrome, 0.06 (95% CI, 0.02–0.18) vs. 0.02 (95% CI, 0.01–0.03) (IRR, 2.88; 95% CI, 0.93–8.93); and dysthymia, 0.12 (95% CI, 0.05–0.26) vs. 0.16 (95% CI, 0.14–0.19) (IRR, 0.71; 95% CI, 0.32–1.57). Detailed IRs and IRRs by sex, age group, and surgery status are presented in Table 2 and Supplementary Tables S1–S9.

### 3.3. Risk Factors for Psychiatric Disorders by Cox Proportional Hazard Model

The Cox proportional hazards model assessed the adjusted risk of psychiatric disorders, including overall disorders, depression, anxiety, stress-related and adjustment disorders, conduct disorder, HKD, autism, Asperger syndrome, and dysthymia. Age group, sex, income level, and microtia status (overall, without surgery, with surgery) were evaluated as risk factors. Compared with the 0- to 4-year group, HRs for overall disorders decreased with age: 0.84 (95% CI, 0.78–0.90) for 5–9 years, 0.77 (95% CI, 0.71–0.82) for 10–14 years, and 0.70 (95% CI, 0.63–0.77) for 15–19 years. Female sex increased risk (HR, 1.33; 95% CI, 1.27–1.40), while middle- and high-income levels were protective (HRs, 0.85 [95% CI, 0.79–0.91] and 0.93 [95% CI, 0.87–1.00], respectively) vs. low income.

Microtia status was a significant risk factor for several disorders: overall disorders (HR, 1.20; 95% CI, 1.07–1.34), with surgery (HR, 1.31; 95% CI, 1.10–1.57) and without (HR, 1.14; 95% CI, 0.99–1.31). Depression (HR, 0.55; 95% CI, 0.44–0.68) and anxiety (HR, 0.69; 95% CI, 0.54–0.87) showed lower risk, with surgery-associated HRs of 0.72 and 0.71, similar to non-surgery. Stress-related and adjustment disorders (HR, 0.85; 95% CI, 0.63–1.15) and dysthymia (HR, 0.83; 95% CI, 0.37–1.89) had no significant association. Conduct disorder (HR, 1.84; 95% CI, 0.93–3.64) and Asperger syndrome (HR, 2.69; 95% CI, 0.75–9.56) showed elevated but non-significant risks. HKD (HR, 5.58; 95% CI, 4.62–6.74) and autism (HR, 9.90; 95% CI, 4.85–20.22) were strongly associated (Table 3).

### 3.4. Subgroup Analyses for Risk of Psychiatric Disorders in Patients with Microtia

**Figure 2:**
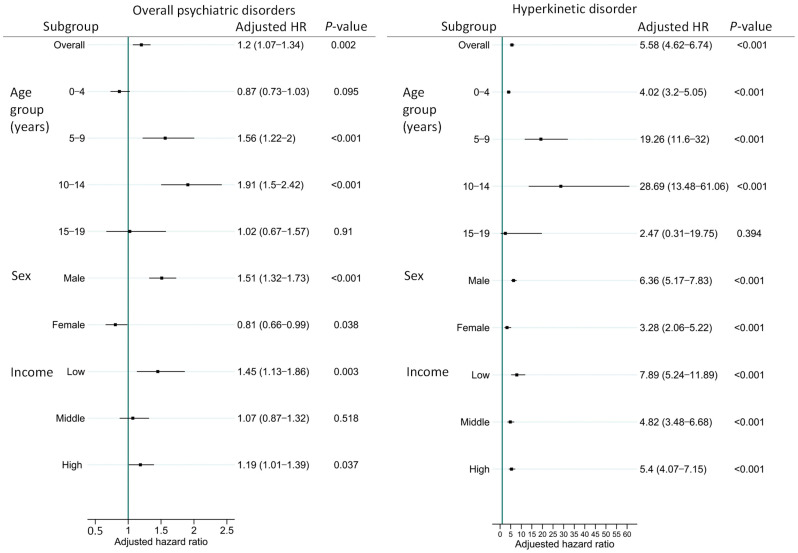
Forest plots of risk for overall psychiatric disorders and hyperkinetic disorder (HKD) in microtia subgroups stratified by age group, sex, and income level. HRs were derived from multivariate Cox proportional hazards models adjusted for age group, sex, and income level. The reference group comprised age- and sex-matched controls without microtia. HR, hazard ratio; CI, confidence interval; HKD, hyperkinetic disorder.

A subgroup analysis was conducted using the multivariate Cox proportional hazards model for overall psychiatric disorders and HKD, where microtia was identified as a risk factor. Stratified analyses were performed based on age group, sex, and income level. For overall psychiatric disorders, the microtia cohort exhibited an elevated risk in the 5- to 9-year and 10- to 14-year age groups, among males, and in the low-income group. For HKD, the microtia cohort showed an increased risk across all subgroups except the 15- to 19-year age group (Figure 2).

### 3.5. Association Between Ear Reconstruction Surgery and Risk of Psychiatric Disorders in Patients with Microtia

To evaluate the association between ear reconstruction surgery and psychiatric disorders where microtia showed an independent association, we compared patients with microtia who underwent ear reconstruction surgery with those who did not, focusing on overall psychiatric disorders and HKD. Autism was excluded from statistical analysis due to an insufficient number of surgically treated patients.

**Figure 3:**
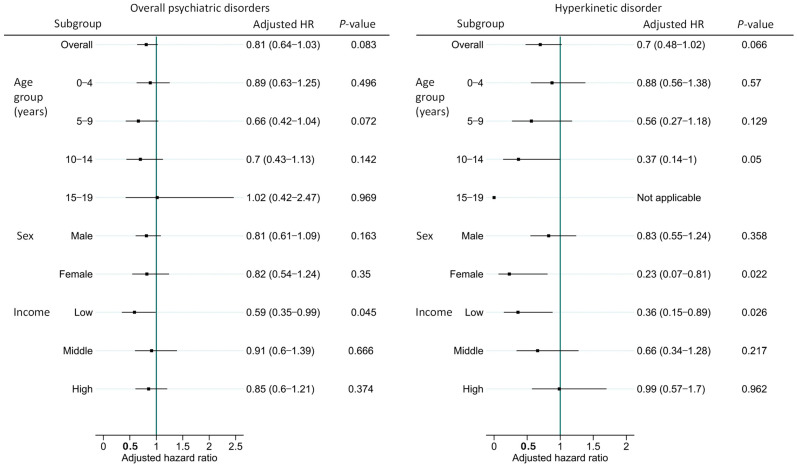
Forest plots depicting adjusted hazard ratios (HRs) and 95% confidence intervals (CIs) for overall psychiatric disorders and hyperkinetic disorder (HKD) in patients with microtia, stratified by ear reconstruction surgery status and further stratified by age group, sex, and income level. HRs were derived from multivariate Cox proportional hazards models adjusted for age group, sex, and income level, with the non-surgery microtia group as the reference. HR, hazard ratio; CI, confidence interval; HKD, hyperkinetic disorder.

Overall, surgery was not significantly associated with a lower risk of overall psychiatric disorders; however, it was associated with a significantly lower risk among patients with low socioeconomic status. Similarly, surgery was not associated with a lower overall risk of HKD, but it was associated with a significantly lower HKD risk among patients aged 10 to 14 years, female patients, and those with low socioeconomic status (Figure 3).

## 4. Discussion

In this nationwide cohort study, we evaluated the risks of psychiatric disorders, including depression, anxiety, stress-related and adjustment disorders, conduct disorder, HKD, autism, Asperger syndrome, and dysthymia, in pediatric and adolescent patients with microtia. We found that microtia was associated with an increased risk of overall psychiatric disorders, particularly HKD and autism. Subgroup analyses further highlighted the demographic vulnerabilities for overall psychiatric disorders and HKD.

Several studies have investigated the psychiatric impact of microtia. Steffen et al. [16] reported improved psychosocial outcomes following ear reconstruction with rib cartilage, assessed using the Frankfurter Selbstkonzeptskalen (Frankfurt Self-concept Scales; FSKN) questionnaire. Jiamei et al. [6] conducted a psychiatric analysis of patients with congenital microtia using the Symptom Checklist-90 (SCL-90) and Child Behavior Checklist, finding that these patients exhibited depression, interpersonal sensitivity/social difficulties, and hostility/aggression. Johns et al. [17] evaluated the psychiatric status of 23 patients in the United States, scoring their profiles with the Behavioral Assessment System for Children-Second Edition. However, these studies did not assess patients who had received actual psychiatric care but instead relied on questionnaire-based evaluations. Additionally, as single-center studies from specific hospitals, they involved small sample sizes and produced results that may not reflect real-world conditions, thus conferring a low level of evidence. Although these studies reported elevated depression and anxiety moods among patients with microtia, our population-based study adopted a more stringent definition, limiting these conditions to those formally diagnosed by psychiatrists and receiving psychotherapies. Consequently, the adjusted hazard ratios for depressive disorder (HR, 0.55) and anxiety disorder (HR, 0.69) in the microtia cohort were lower than those in the control group. Several methodological and clinical factors may account for this counterintuitive observation. First, our stringent outcome definition, requiring both an ICD-10 diagnostic code and psychotherapy administration, may set a threshold that excludes subclinical presentations not necessitating formal psychotherapy. Second, patients with microtia primarily receive care from otolaryngologists and plastic surgeons, specialties in which concurrent mood and anxiety symptoms may be underrecognized. Third, approximately 59% of the microtia cohort was aged 0–4 years at enrollment, and depressive and anxiety disorders are more commonly diagnosed during adolescence; thus, the follow-up period may not have captured the peak incidence years for these disorders. Fourth, clinicians may prioritize neurodevelopmental diagnoses such as HKD or autism over comorbid mood disorders when patients with microtia are referred for psychiatric evaluation. Finally, the heightened medical surveillance inherent in reconstructive and audiological care may preferentially detect externalizing rather than internalizing conditions. Accordingly, the lower hazard ratios should not be interpreted as evidence of a protective effect; rather, they likely reflect the combined effects of a restrictive outcome definition, age-related diagnostic patterns, and differential clinical attention.

Although the mechanisms underlying the association between microtia and HKD or autism were not directly tested in the present study, the existing literature suggests several plausible hypotheses involving genetic and prenatal environmental factors. As a genetic factor, predispositions, including copy number variations (CNVs) and de novo mutations (e.g., HOXA2, SHANK2), have been implicated in microtia and autism, respectively [18,19,20]. Similarly, variants in DRD4 and CDH13 are associated with HKD, indicating a spectrum of neurodevelopmental genetic risks [21,22].

Prenatal environmental factors may further contribute to this association. Maternal conditions such as diabetes, exposure to teratogens (e.g., retinoic acid), and prenatal stress have been linked to microtia [3]; advanced maternal age and obstetric complications are risk factors for autism [23]; and prenatal stressors may contribute to basal ganglia dysfunction, a key feature of HKD [21]. The convergence of these environmental insults during critical periods of fetal development—particularly affecting neural tube closure and craniofacial patterning—suggests a common pathway that could predispose individuals to multiple neurodevelopmental phenotypes. Early neurodevelopmental anomalies provide an additional layer of connectivity. Microtia’s origin in the defective formation of the auricle reflects disruptions in the same developmental window as the emergence of synaptic and mitochondrial dysfunctions in autism and basal ganglia impairments in HKD [24,25]. The overlap in the timing of these anomalies, particularly during the first trimester, supports the notion that a shared insult—whether genetic or environmental—could simultaneously affect ear morphogenesis and brain connectivity. These putative mechanisms, while supported by biological plausibility, were not directly examined in the present study and warrant future investigation.

HKD, akin to attention-deficit/hyperactivity disorder (ADHD), is influenced by genetic factors as well as environmental factors. Environmental contributors include social stress (e.g., stigma, bullying, and social exclusion), reduced self-esteem (e.g., negative self-perception due to appearance), and resource scarcity (e.g., limited access to mental health support or education, particularly in low-income groups) [26,27,28,29]. Patients with microtia, characterized by a visible ear deformity, are prone to negative attention in school or community settings, potentially leading to stigma and bullying. This effect may be amplified in low-income populations with fewer resources to mitigate such stressors. The observed association between ear reconstruction surgery and a lower HKD risk in specific subgroups may hypothetically reflect the alleviation of appearance-related psychosocial stressors through improved appearance, reduced social stigma, and enhanced self-esteem. These changes may hypothetically contribute to a lower incidence or severity of HKD, especially in populations where appearance significantly affects social dynamics. The observed associations indicate that specific subgroups—patients aged 10 to 14 years, females, and those with low socioeconomic status—exhibited a lower HKD risk. This finding may be attributable to heightened vulnerability to appearance-related stress during adolescence, when peer acceptance is critical, and in females, who face greater societal pressure regarding aesthetics. In low-income patients, limited access to mental health resources exacerbates stress, making surgical intervention a potentially relevant factor associated with lower HKD risk through the attenuation of environmental triggers.

In addition to the appearance-related and psychosocial stressors, the functional audiological characteristics of microtia play a crucial role in the development of HKD symptoms. Microtia is frequently accompanied by congenital aural atresia, leading to unilateral or bilateral conductive hearing loss. Children with conductive hearing loss must exert significant ‘listening effort’ to process speech and auditory information in noisy environments, such as classrooms. This continuous audiological strain rapidly leads to subjective and cognitive fatigue [30]. Chronic cognitive fatigue can easily manifest as inattention, distractibility, and secondary hyperactivity, which strongly mimic or exacerbate the core clinical features of HKD [31]. Therefore, the elevated risk of HKD in patients with microtia should be understood as a complex interplay between psychosocial vulnerabilities due to appearance and cognitive overloads caused by hearing impairment.

One limitation of our research was the reliance on ICD-10 coding for diagnostic categorization, which lacked the granularity to assess severity or the specific type of microtia. Furthermore, the absence of laboratory data, medical imaging, or photographic evidence may have contributed to potential biases in our diagnostic classifications. However, the Korean NHI system offers reliable data, as ICD-10 codes are validated by the insurance review committees of individual general hospitals prior to medical fee claims. Additionally, the Health Insurance Review & Assessment Service, a governmental body in Korea, conducts further validation and maintains the NHI dataset following re-evaluation [9,10,11,12,13,14]. We also assessed the diagnostic precision of the ICD-10 coding system, which demonstrated high sensitivity and specificity, suggesting a minimal likelihood of misclassification bias. Another limitation is that the socioeconomic status variable used in this study represents an income-based proxy derived from NHIS premium tiers. It does not incorporate other dimensions of socioeconomic position, such as educational attainment, occupation, or household size. Non-equivalized income may not be directly comparable across families of different sizes, and unmeasured socioeconomic factors may confound both the likelihood of undergoing ear reconstruction surgery and mental health outcomes. Finally, although our surgical subgroup analysis was designed to mitigate immortal time bias by resetting the time origin to the surgery date, formal time-varying covariate modeling may provide additional methodological rigor in future studies.

Despite the limitations, one of the strengths of our study is that it is the largest investigation of associations between microtia and psychiatric disorders, to the best of our knowledge. With a large sample size, our study had sufficient power to explore the risk of psychiatric disorders in patients with microtia by age and sex. Furthermore, we conducted an incidence-case–cohort study with a sufficient follow-up duration to observe changes in cumulative incidence and the risk of psychiatric disorders in patients with microtia. Finally, as a population-based cohort study, we captured real-world clinical effectiveness rather than theoretical efficacy, with results reflecting real-world conditions.

## 5. Conclusions

Microtia is associated with an increased risk of psychiatric disorders in pediatric and adolescent populations, particularly HKD and childhood autism. These elevated risks were concentrated among individuals aged 5 to 14 years, males, and those with a low socioeconomic status. These findings underscore the importance of early psychiatric screening and multidisciplinary care for children with microtia.

## Figures and Tables

**Table 1:** Baseline characteristics of the study population.

Characteristics	Total Population		Male Subjects		Female Subjects	
	Microtia (*n* = 6048), *n* (%)	Control (*n* = 120,960), *n* (%)	Microtia (*n* = 3789), *n* (%)	Control (*n* = 75,780), *n* (%)	Microtia (*n* = 2259), *n* (%)	Control (*n* = 45,180), *n* (%)
**Age (years)**						
0–4	3566 (58.96)	71,320 (58.96)	2203 (58.14)	44,060 (58.14)	1363 (60.34)	27,260 (60.34)
5–9	984 (16.27)	19,680 (16.27)	616 (16.26)	12,320 (16.26)	368 (16.29)	7360 (16.29)
10–14	934 (15.44)	18,680 (15.44)	593 (15.65)	11,860 (15.65)	341 (15.10)	6820 (15.10)
15–19	564 (9.33)	11,280 (9.33)	377 (9.95)	7540 (9.95)	187 (8.28)	3740 (8.28)
**Sex**						
Male	3789 (62.65)	75,780 (62.65)	3789 (62.65)	75,780 (62.65)	—	—
Female	2259 (37.35)	45,180 (37.35)	—	—	2259 (37.35)	45,180 (37.35)
**Income level**						
Low	974 (16.11)	19,355 (16.00)	613 (16.18)	12,101 (15.97)	361 (15.98)	7254 (16.06)
Middle	1970 (32.57)	51,081 (42.23)	1243 (32.81)	31,811 (41.98)	727 (32.18)	19,270 (42.65)
High	2912 (48.15)	48,312 (39.94)	1818 (47.98)	30,489 (40.23)	1094 (48.43)	17,823 (39.45)
Missing	192 (3.17)	2212 (1.83)	115 (3.04)	1379 (1.82)	77 (3.41)	833 (1.84)
**Microtia surgery**						
No	4270 (70.60)	N/A	2619 (69.12)	N/A	1651 (73.09)	N/A
Yes	1778 (29.40)	N/A	1170 (30.88)	N/A	608 (26.91)	N/A

N/A, not applicable.

**Table 2:** Summarizing incidence rates of each psychiatric disorder in the microtia cohort and the control cohort.

	Microtia Cohort (With or Without Surgery)		Control Cohort		
Outcome Disorders	Events/PYS	Incidence Rate ^a^ (95% CI)	Events/PYS	Incidence Rate ^a^ (95% CI)	IRR (95% CI)
Overall psychiatric disorder	358/51,758.23	6.92 (6.24–7.67)	6203/1,043,060.9	5.95 (5.80–6.10)	1.16 (1.05–1.29)
Depressive disorder	108/51,758.23	2.09 (1.73–2.52)	3685/1,043,060.9	3.53 (3.42–3.65)	0.59 (0.49–0.71)
Anxiety disorder	85/51,758.23	1.64 (1.33–2.03)	2460/1,043,060.9	2.36 (2.27–2.45)	0.70 (0.56–0.86)
Stress-related and adjustment disorders	49/51,758.23	0.95 (0.72–1.25)	1197/1,043,060.9	1.15 (1.08–1.21)	0.82 (0.62–1.09)
Conduct disorder	10/51,758.23	0.19 (0.10–0.36)	111/1,043,060.9	0.11 (0.09–0.13)	1.82 (0.98–3.37)
Hyperkinetic disorder	152/51,758.23	2.94 (2.51–3.44)	573/1,043,060.9	0.55 (0.51–0.60)	5.35 (4.56–6.27)
Autism	12/51,758.23	0.23 (0.13–0.41)	25/1,043,060.9	0.02 (0.02–0.04)	9.67 (5.49–17.03)
Asperger syndrome	3/51,758.23	0.06 (0.02–0.18)	21/1,043,060.9	0.02 (0.01–0.03)	2.88 (0.93–8.93)
Dysthymia	6/51,758.23	0.12 (0.05–0.26)	171/1,043,060.9	0.16 (0.14–0.19)	0.71 (0.32–1.57)

CI, 95% confidence interval; IRR, incidence rate ratio; PYS, person-years. ^a^ Per 1000 person-years.

**Table 3:** Adjusted risk for each psychiatric disorder by Cox proportional hazard model.

	**Overall Psychiatric Disorders**			**Depressive Disorders**			**Anxiety Disorders**		
**Risk Factors**	**HR**	**95% CI**	***p***	**HR**	**95% CI**	***p***	**HR**	**95% CI**	***p***
**Age group (years)**									
0–4 (ref)	1	ref	—	1	ref	—	1	ref	—
5–9	0.84	0.78–0.90	<0.001	0.85	0.78–0.94	0.001	1.05	0.95–1.17	0.355
10–14	0.77	0.71–0.82	<0.001	0.75	0.68–0.82	<0.001	0.98	0.88–1.09	0.706
15–19	0.70	0.63–0.77	<0.001	0.67	0.59–0.76	<0.001	0.91	0.79–1.05	0.203
Female (vs. male)	1.33	1.27–1.40	<0.001	1.59	1.49–1.70	<0.001	1.39	1.28–1.50	<0.001
**Income level**									
Low (ref)	1	ref	—	1	ref	—	1	ref	—
Middle	0.85	0.79–0.91	<0.001	0.84	0.77–0.92	<0.001	0.86	0.77–0.97	0.010
High	0.93	0.87–1.00	0.051	0.92	0.84–1.01	0.081	0.94	0.84–1.05	0.251
**Microtia (vs. control)**									
All	1.20	1.07–1.34	0.002	0.55	0.44–0.68	<0.001	0.69	0.54–0.87	0.002
Without surgery	1.14	0.99–1.31	0.069	0.46	0.35–0.61	<0.001	0.67	0.51–0.89	0.006
With surgery	1.31	1.10–1.57	0.003	0.72	0.53–0.98	0.039	0.71	0.50–1.03	0.069
	**Stress-Related/Adjustment Disorders**			**Conduct Disorder**			**Hyperkinetic Disorder**		
**Risk Factors**	**HR**	**95% CI**	***p***	**HR**	**95% CI**	***p***	**HR**	**95% CI**	***p***
**Age group (years)**									
0–4 (ref)	1	ref	—	1	ref	—	1	ref	—
5–9	0.75	0.64–0.89	0.001	0.26	0.13–0.54	<0.001	0.32	0.25–0.41	<0.001
10–14	0.74	0.63–0.88	0.001	0.26	0.13–0.54	<0.001	0.13	0.09–0.19	<0.001
15–19	0.70	0.56–0.86	0.001	N/A	N/A	N/A	0.08	0.04–0.16	<0.001
Female (vs. male)	1.22	1.08–1.36	0.001	0.60	0.40–0.90	0.013	0.53	0.45–0.63	<0.001
**Income level**									
Low (ref)	1	ref	—	1	ref	—	1	ref	—
Middle	0.77	0.66–0.90	0.001	0.83	0.49–1.40	0.482	0.75	0.61–0.92	0.007
High	0.96	0.82–1.12	0.572	0.97	0.57–1.63	0.899	0.83	0.67–1.02	0.075
**Microtia (vs. control)**									
All	0.85	0.63–1.15	0.294	1.84	0.93–3.64	0.079	5.58	4.62–6.74	<0.001
Without surgery	0.69	0.46–1.03	0.069	1.62	0.71–3.70	0.251	5.42	4.37–6.71	<0.001
With surgery	1.19	0.77–1.86	0.432	2.53	0.80–8.02	0.115	6.06	4.40–8.34	<0.001
	**Autism**			**Asperger Syndrome**			**Dysthymia**		
**Risk Factors**	**HR**	**95% CI**	***p***	**HR**	**95% CI**	***p***	**HR**	**95% CI**	***p***
**Age group (years)**									
0–4 (ref)	1	ref	—	1	ref	—	1	ref	—
5–9	0.81	0.33–1.98	0.641	0.13	0.02–0.99	0.049	1.26	0.86–1.84	0.229
10–14	0.46	0.14–1.53	0.204	0.22	0.05–1.01	0.051	0.80	0.51–1.26	0.341
15–19	0.20	0.03–1.48	0.115	N/A	N/A	N/A	0.70	0.38–1.27	0.239
Female (vs. male)	0.47	0.21–1.03	0.058	0.59	0.23–1.50	0.271	1.61	1.19–2.17	0.002
**Income level**									
Low (ref)	1	ref	—	1	ref	—	1	ref	—
Middle	0.78	0.30–2.06	0.619	0.68	0.17–2.71	0.580	0.88	0.56–1.38	0.580
High	1.03	0.40–2.61	0.956	1.87	0.54–6.50	0.327	1.20	0.78–1.85	0.414
**Microtia (vs. control)**									
All	9.90	4.85–20.22	<0.001	2.69	0.75–9.56	0.127	0.83	0.37–1.89	0.664
Without surgery	14.48	7.04–29.77	<0.001	1.26	0.17–9.56	0.825	0.64	0.20–2.00	0.441
With surgery	N/A	N/A	N/A	6.74	1.43–31.71	0.016	1.21	0.38–3.80	0.747

CI, 95% confidence interval; ref, reference; HR, hazard ratio; N/A, not applicable.

## Data Availability

The data used in this study are from the Korean National Health Insurance Service (NHIS) database. Data are not publicly available as they are owned by the NHIS and access is restricted. Researchers may apply for access through the NHIS data sharing system (https://nhiss.nhis.or.kr, accessed on 15 April 2026).

## Supplementary Materials

The following supporting information can be downloaded at: https://www.mdpi.com/article/10.3390/jcm15082998/s1, Figures S1–S9: Kaplan–Meier curves for cumulative incidence of each psychiatric disorder; Tables S1–S9: Detailed IRs and IRRs by sex, age group, and surgery status for each psychiatric disorder.

## Abbreviations

The following abbreviations are used in this manuscript:

ADHD	Attention-deficit/hyperactivity disorder
CI	Confidence interval
CNV	Copy number variation
FSKN	Frankfurter Selbstkonzeptskalen (Frankfurt Self-concept Scales)
HKD	Hyperkinetic disorder
HR	Hazard ratio
ICD-10	International Classification of Diseases, Tenth Revision
IR	Incidence rate
IRB	Institutional Review Board
IRR	Incidence rate ratio
NHI	National Health Insurance
NHIS-INCHS	National Health Insurance Service-Infants and Children’s Health Screening
PYS	Person-years
SCL-90	Symptom Checklist-90
