# Supplementary material for: Time-Dependent Risk of Psychiatric Disorders in Pediatric and Adolescent Patients with Microtia: A Nationwide Population-Based Cohort Study
Source: J Clin Med. 2026 Apr 15;15(8):2998. doi: 10.3390/jcm15082998 (PMC13116245; doi:10.3390/jcm15082998)

Supplementary Figure S1. Kaplan–Meier curves for the incidence of overall psychiatric disorders in patients with microtia and the control cohort.

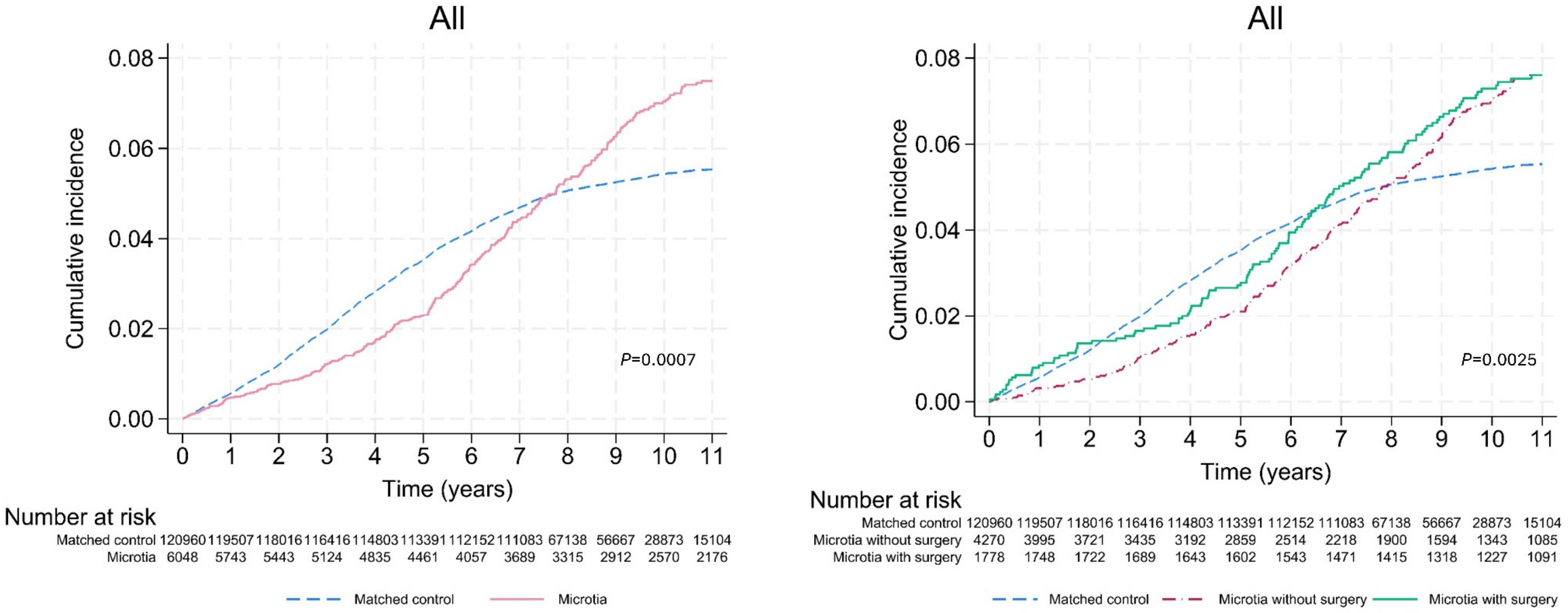

Supplementary Figure S2. Kaplan–Meier curves for the incidence of depression disorder in patients with microtia and the control cohort.

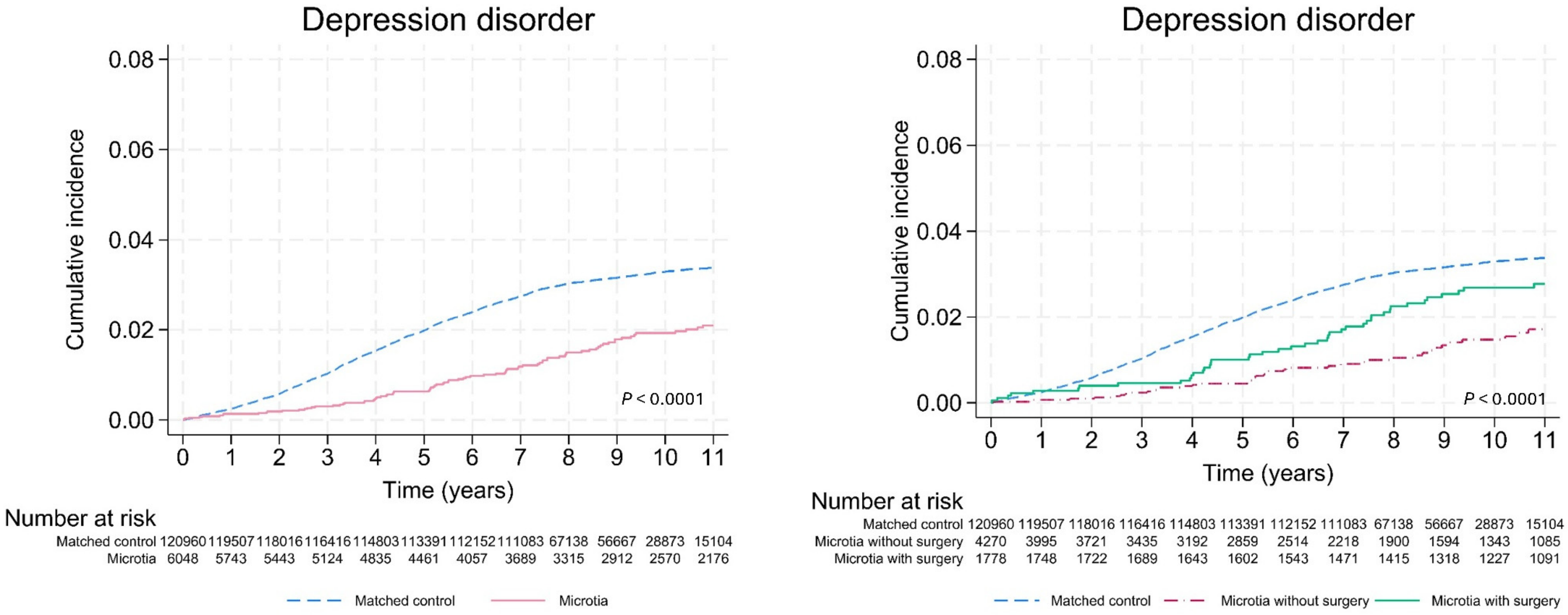

Supplementary Figure S3. Kaplan–Meier curves for the incidence of anxiety disorder in patients with microtia and the control cohort.

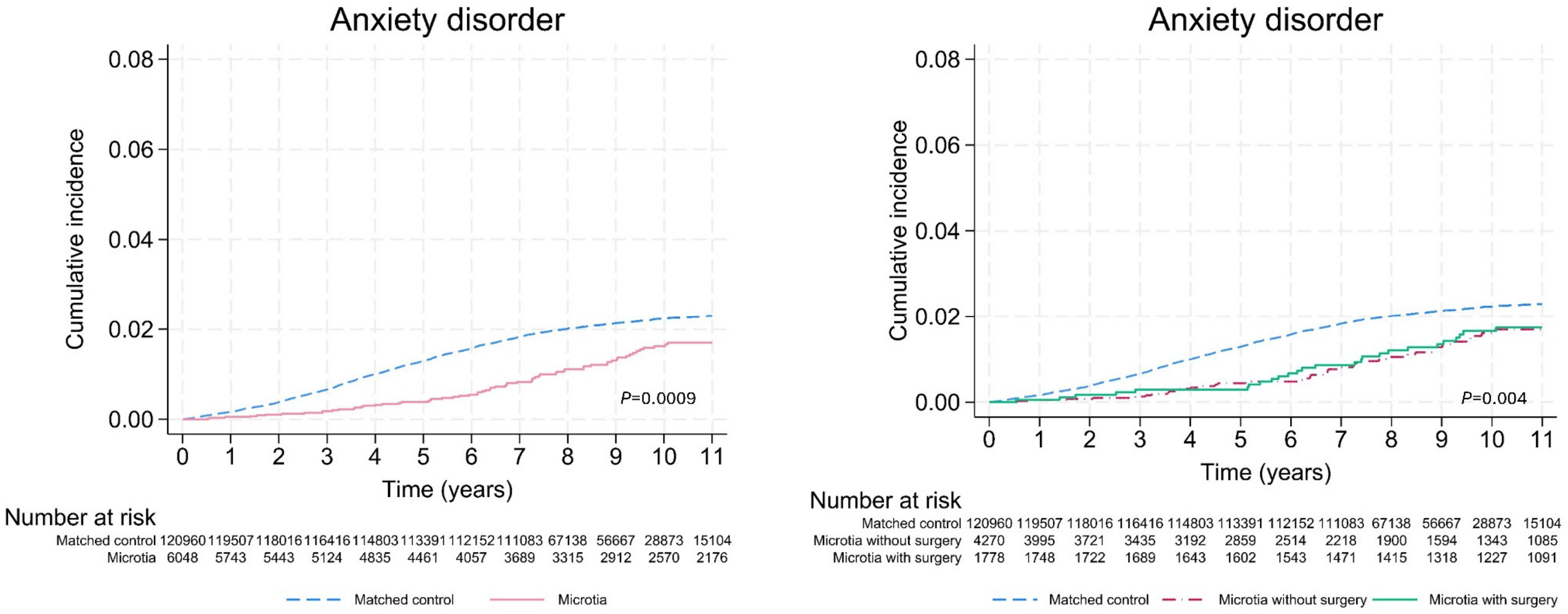

Supplementary Figure S4. Kaplan–Meier curves for the incidence of stress-related and adjustment disorders in patients with microtia and the control cohort.

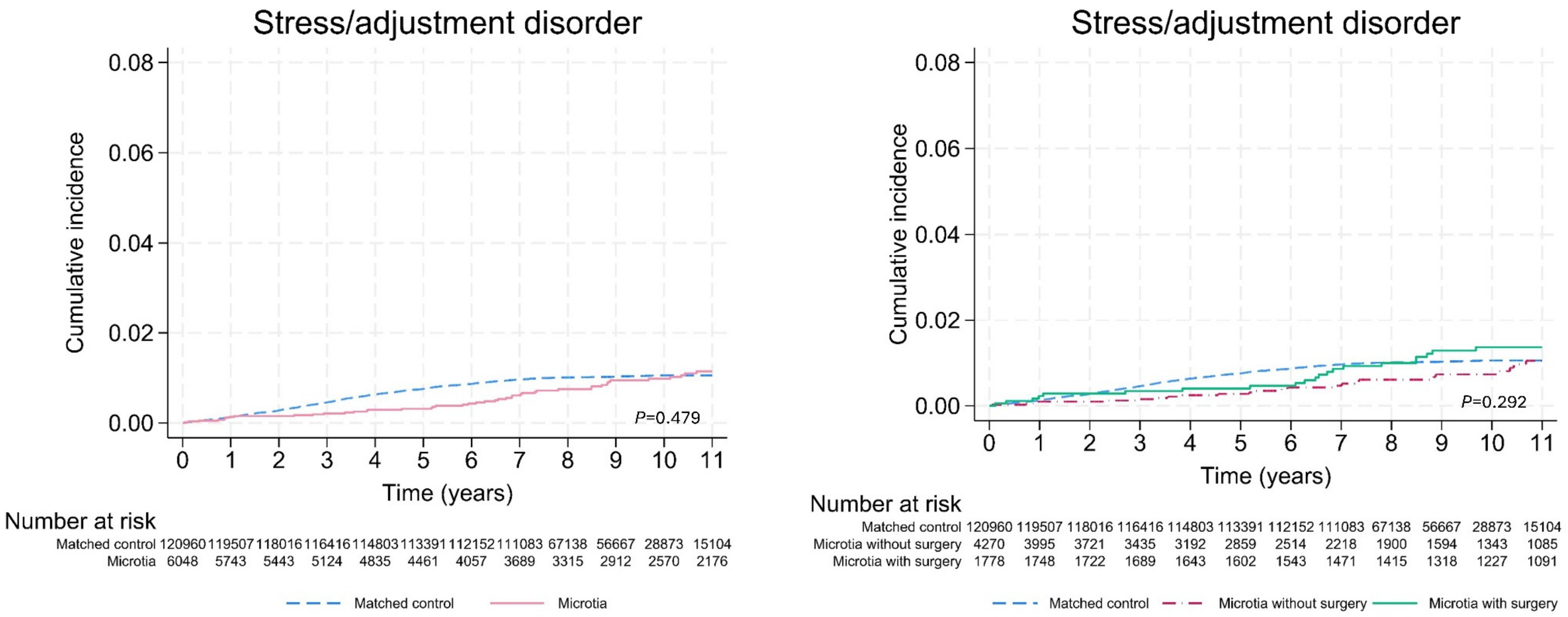

Supplementary Figure S5. Kaplan–Meier curves for the incidence of conduct disorder in patients with microtia and the control cohort.

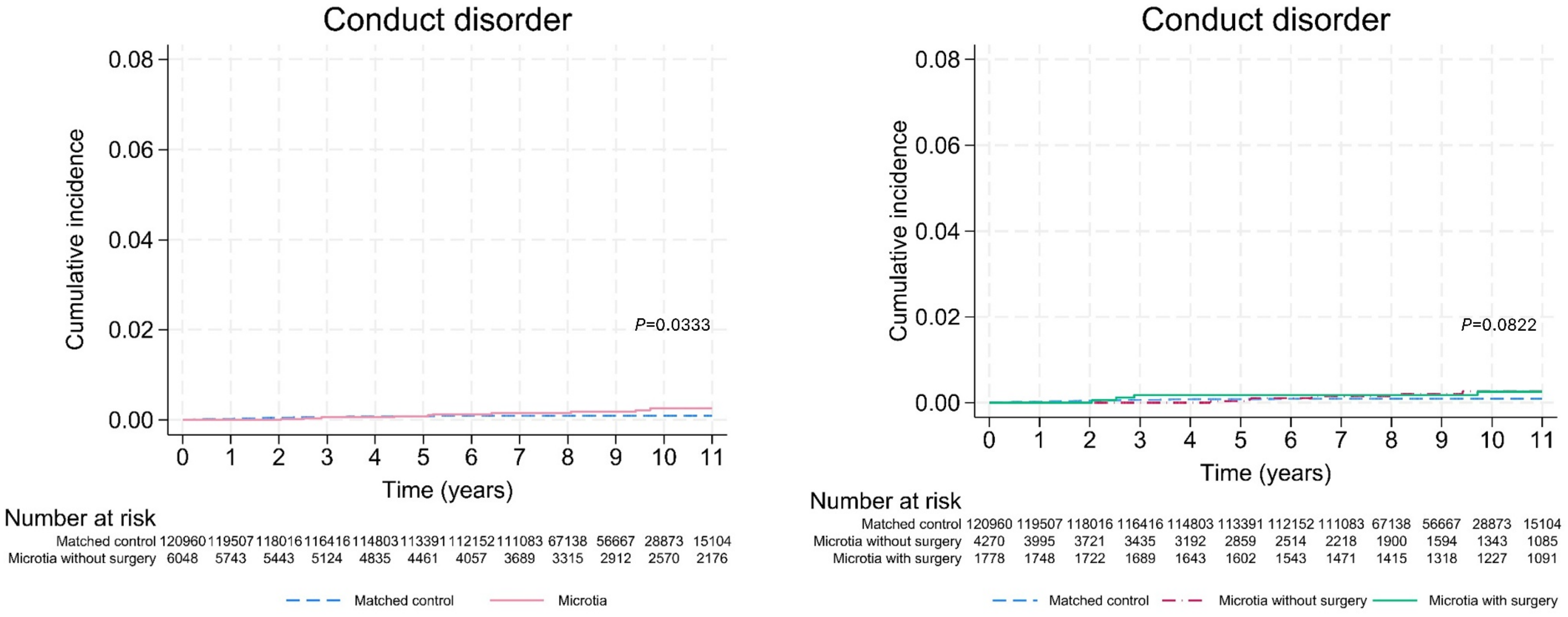

Supplementary Figure S6. Kaplan–Meier curves for the incidence of hyperkinetic disorder in patients with microtia and the control cohort.

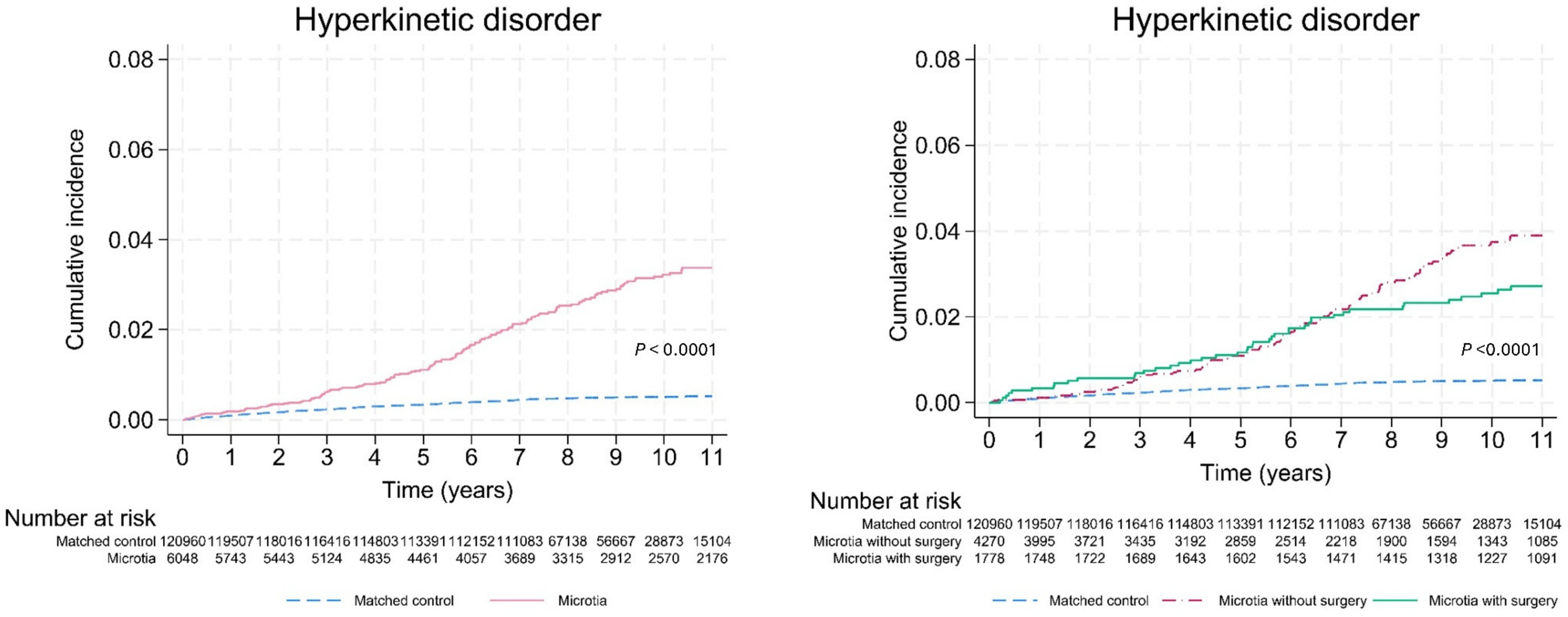

Supplementary Figure S7. Kaplan–Meier curves for the incidence of autism in patients with microtia and the control cohort.

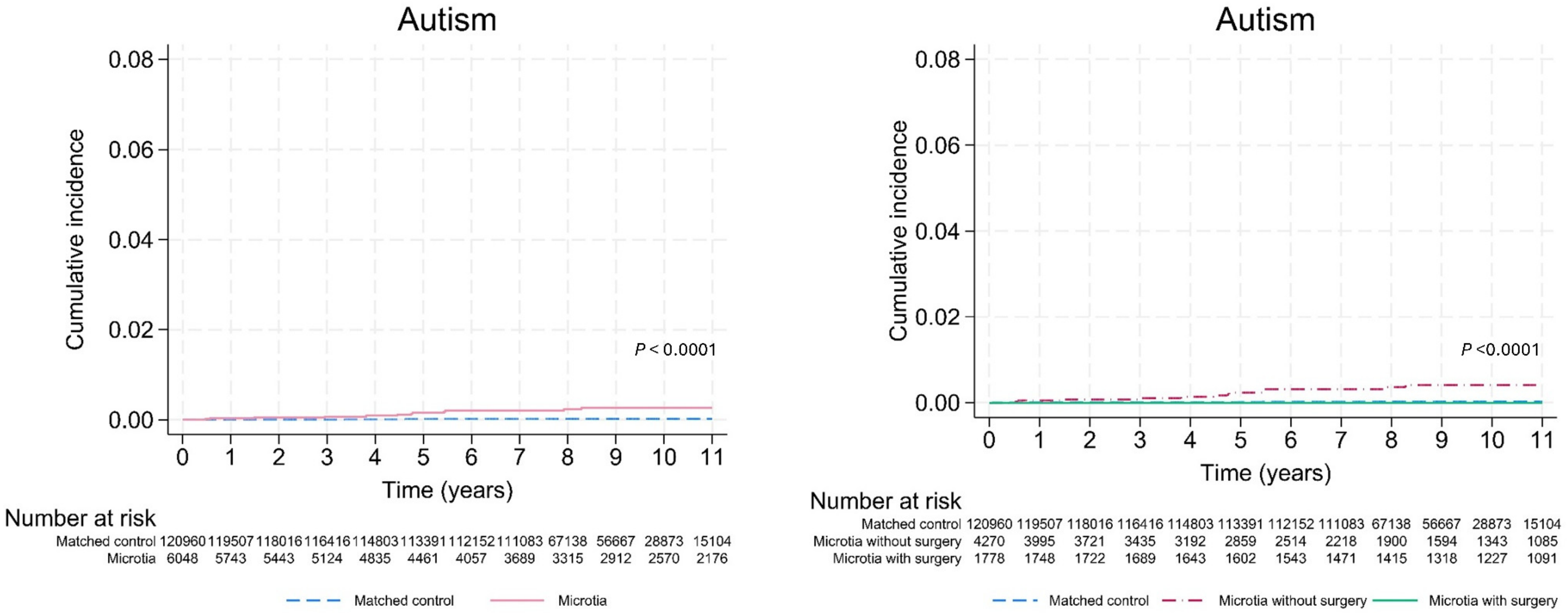

Supplementary Figure S8. Kaplan–Meier curves for the incidence of Asperger syndrome in patients with microtia and the control cohort.

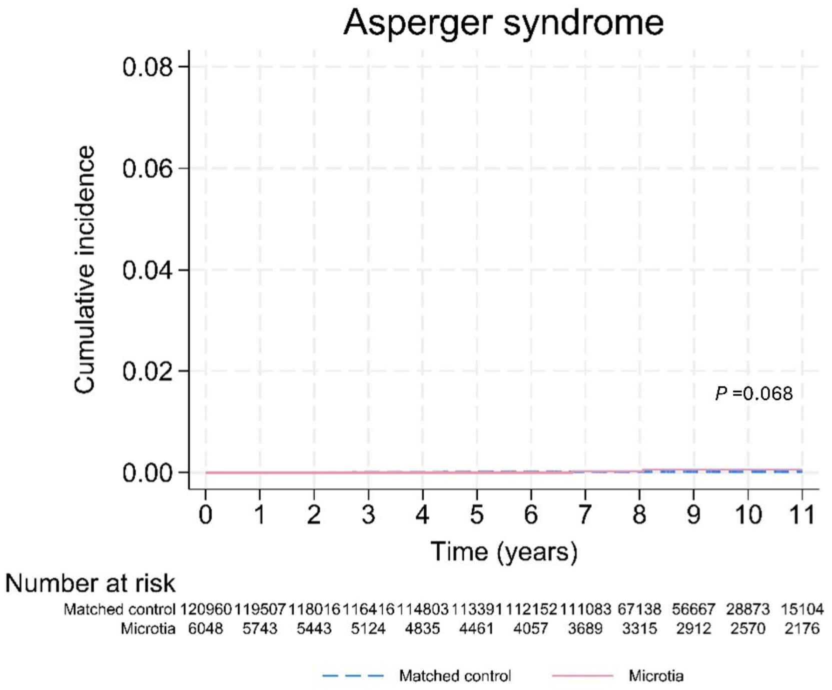

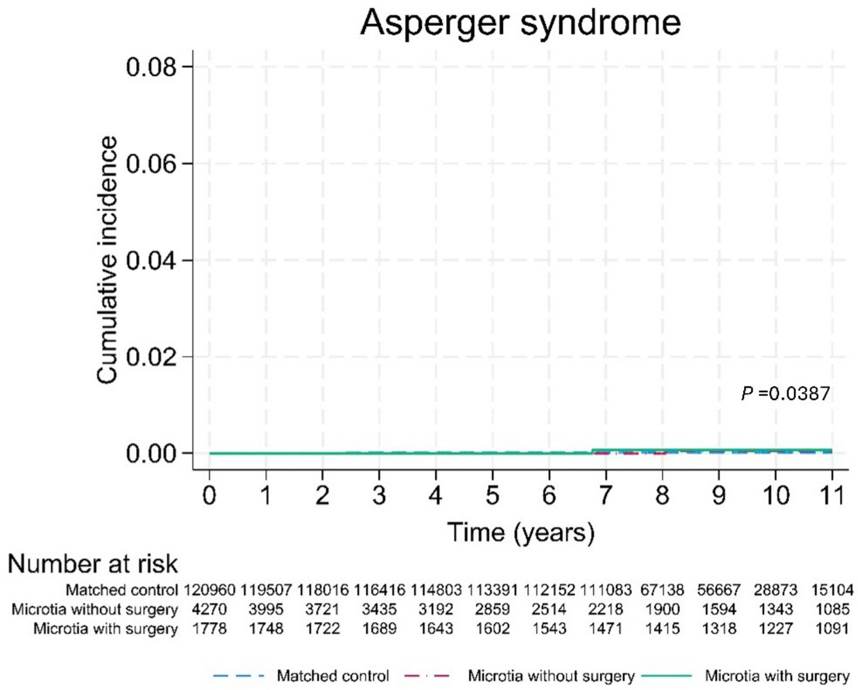

Supplementary Figure S9. Kaplan–Meier curves for the incidence of dysthymia in patients with microtia and the control cohort.

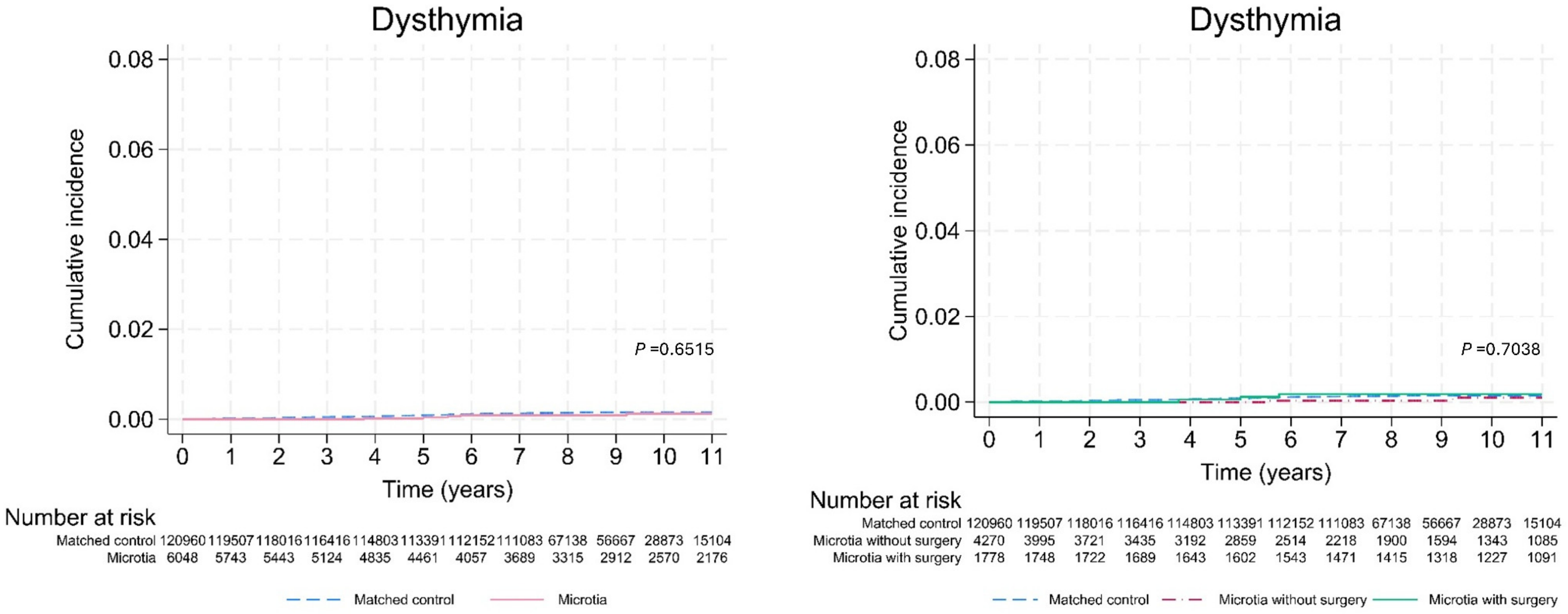

Supplement: Supplementary file 1 [file jcm-15-02998-s001.zip › Supplementary figures.pdf]
