# Supplementary material for: Time-Dependent Risk of Psychiatric Disorders in Pediatric and Adolescent Patients with Microtia: A Nationwide Population-Based Cohort Study
Source: J Clin Med. 2026 Apr 15;15(8):2998. doi: 10.3390/jcm15082998 (PMC13116245; doi:10.3390/jcm15082998)
Supplement: Supplementary file 1 [file jcm-15-02998-s001.zip › Supplementary tables.pdf]

Supplementary Table S1. Incidence Rates of Overall Psychiatric Disorder in the Microtia Cohort and the Control Cohort.

		**All microtia with or without surgery**			**Control cohort**		
**Outcome**	**Age/sex**	**Events/PYS**	**Incidence rate[a] (CI)**	**IRR (CI)**	**Events/PYS**	**Incidence rate[a] (CI)**	**IRR (CI)**
Overall psychiatric disorders	All	358/51758.23	6.92 (6.24–7.67)	1.16 (1.05–1.29)	6203/1043060.9	5.95 (5.8–6.1)	1 (0.98–1.03)
	Male	242/32597.83	7.42 (6.54–8.42)	1.42 (1.25–1.61)	3446/660234.36	5.22 (5.05–5.4)	1 (0.97–1.03)
	Female	116/19160.4	6.05 (5.05–7.26)	0.84 (0.7–1.01)	2757/382826.54	7.2 (6.94–7.48)	1 (0.96–1.04)
	Age group						
	0–4 years	178/27893.54	6.38 (5.51–7.39)	0.9 (0.78–1.05)	3892/551169.75	7.06 (6.84–7.29)	1 (0.97–1.03)
	5–9 years	81/9129.61	8.87 (7.14–11.03)	1.72 (1.39–2.14)	958/186012.81	5.15 (4.83–5.49)	1 (0.94–1.07)
	10–14 years	75/9407.09	7.97 (6.36–10)	1.81 (1.44–2.26)	881/199551.1	4.41 (4.13–4.72)	1 (0.94–1.07)
	15–19 years	24/5327.99	4.5 (3.02–6.72)	1.01 (0.68–1.51)	472/106327.25	4.44 (4.06–4.86)	1 (0.91–1.09)
		**Microtia without surgery**			**Microtia with surgery**		
	**Age/sex**	**Events/PYS**	**Incidence rate[a] (CI)**	**IRR (CI)**	**Events/PYS**	**Incidence rate[a] (CI)**	**IRR (CI)**
	All	221/31986.85	6.91 (6.06–7.88)	1.16 (1.02–1.33)	137/19771.38	6.93 (5.86–8.19)	1.17 (0.99–1.38)
	Male	150/19583.94	7.66 (6.53–8.99)	1.47 (1.25–1.72)	92/13013.88	7.07 (5.76–8.67)	1.35 (1.1–1.66)
	Female	71/12402.9	5.72 (4.54–7.22)	0.79 (0.63–1)	45/6757.5	6.66 (4.97–8.92)	0.92 (0.69–1.24)
	Age group						
	0–4 years	133/21336.56	6.23 (5.26–7.39)	0.88 (0.74–1.05)	45/6556.98	6.86 (5.12–9.19)	0.97 (0.73–1.3)
	5–9 years	42/4122.91	10.19 (7.53–13.78)	1.98 (1.46–2.68)	39/5006.7	7.79 (5.69–10.66)	1.51 (1.11–2.07)
	10–14 years	29/2672.16	10.85 (7.54–15.62)	2.46 (1.71–3.54)	46/6734.93	6.83 (5.12–9.12)	1.55 (1.16–2.07)
	15–19 years	17/3855.21	4.41 (2.74–7.09)	0.99 (0.62–1.6)	7/1472.78	4.75 (2.27–9.97)	1.07 (0.51–2.25)

CI, 95% confidence interval; IRR, incidence rate ratio; PYS, person-years. [a] Per 1000 person-years.

Supplementary Table S2. Incidence Rates of Depression Disorder in the Microtia Cohort and the Control Cohort.

		All microtia with or without surgery			Control cohort		
**Outcome**	**Age/sex**	**Events/PYS**	**Incidence rate[a] (CI)**	**IRR (CI)**	**Events/PYS**	**Incidence rate[a] (CI)**	**IRR (CI)**
Depression disorders	All	108/51758.23	2.09 (1.73–2.52)	0.59 (0.49–0.71)	3685/1043060.9	3.53 (3.42–3.65)	1 (0.97–1.03)
	Male	58/32597.83	1.78 (1.38–2.3)	0.62 (0.48–0.8)	1899/660234.36	2.88 (2.75–3.01)	1 (0.96–1.05)
	Female	50/19160.4	2.61 (1.98–3.44)	0.56 (0.42–0.74)	1786/382826.54	4.67 (4.45–4.89)	1 (0.95–1.05)
	Age group						
	0–4 years	31/27893.54	1.11 (0.78–1.58)	0.26 (0.19–0.37)	2323/551169.75	4.21 (4.05–4.39)	1 (0.96–1.04)
	5–9 years	28/9129.61	3.07 (2.12–4.44)	0.97 (0.67–1.4)	589/186012.81	3.17 (2.92–3.43)	1 (0.92–1.08)
	10–14 years	33/9407.09	3.51 (2.49–4.93)	1.37 (0.97–1.92)	512/199551.1	2.57 (2.35–2.8)	1 (0.92–1.09)
	15–19 years	16/5327.99	3 (1.84–4.9)	1.22 (0.75–2)	261/106327.25	2.45 (2.17–2.77)	1 (0.89–1.13)
		**Microtia without surgery**			**Microtia with surgery**		
	**Age/sex**	**Events/PYS**	**Incidence rate[a] (CI)**	**IRR (CI)**	**Events/PYS**	**Incidence rate[a] (CI)**	**IRR (CI)**
	All	55/31986.85	1.72 (1.32–2.24)	0.49 (0.37–0.63)	53/19771.38	2.68 (2.05–3.51)	0.76 (0.58–0.99)
	Male	29/19583.94	1.48 (1.03–2.13)	0.51 (0.36–0.74)	29/13013.88	2.23 (1.55–3.21)	0.77 (0.54–1.11)
	Female	26/12402.9	2.1 (1.43–3.08)	0.45 (0.31–0.66)	24/6757.5	3.55 (2.38–5.3)	0.76 (0.51–1.14)
	Age group						
	0–4 years	18/21336.56	0.84 (0.53–1.34)	0.2 (0.13–0.32)	13/6556.98	1.98 (1.15–3.41)	0.47 (0.27–0.81)
	5–9 years	11/4122.91	2.67 (1.48–4.82)	0.84 (0.47–1.52)	17/5006.7	3.4 (2.11–5.46)	1.07 (0.67–1.72)
	10–14 years	12/2672.16	3.12 (2.03–4.78)	1.75 (0.99–3.08)	21/6734.93	4.49 (2.55–7.91)	1.22 (0.79–1.86)
	15–19 years	14/3855.21	3.63 (2.15–6.13)	1.48 (0.88–2.5)	2/1472.78	1.36 (0.34–5.43)	0.55 (0.14–2.21)

CI, 95% confidence interval; IRR, incidence rate ratio; PYS, person-years. [a] Per 1000 person-years.

Supplementary Table S3. Incidence Rates of Anxiety Disorder in the Microtia Cohort and the Control Cohort.

		**All microtia with or without surgery**			**Control cohort**		
**Outcome**	**Age/sex**	**Events/PYS**	**Incidence rate[a] (CI)**	**IRR (CI)**	**Events/PYS**	**Incidence rate[a] (CI)**	**IRR (CI)**
Anxiety disorders	All	85/51758.23	1.64 (1.33–2.03)	0.7 (0.56–0.86)	2460/1043060.9	2.36 (2.27–2.45)	1 (0.96–1.04)
	Male	45/32597.83	1.38 (1.03–1.85)	0.67 (0.5–0.89)	1366/660234.36	2.07 (1.96–2.18)	1 (0.95–1.05)
	Female	40/19160.4	2.09 (1.53–2.85)	0.73 (0.54–1)	1094/382826.54	2.86 (2.69–3.03)	1 (0.94–1.06)
	Age group						
	0–4 years	30/27893.54	1.08 (0.75–1.54)	0.43 (0.3–0.61)	1391/551169.75	2.52 (2.39–2.66)	1 (0.95–1.05)
	5–9 years	21/9129.61	2.3 (1.5–3.53)	0.97 (0.63–1.49)	440/186012.81	2.37 (2.15–2.6)	1 (0.91–1.1)
	10–14 years	24/9407.09	2.55 (1.71–3.81)	1.24 (0.83–1.86)	409/199551.1	2.05 (1.86–2.26)	1 (0.91–1.1)
	15–19 years	10/5327.99	1.88 (1.01–3.49)	0.91 (0.49–1.69)	220/106327.25	2.07 (1.81–2.36)	1 (0.88–1.14)
		**Microtia without surgery**			**Microtia with surgery**		
	**Age/sex**	**Events/PYS**	**Incidence rate[a] (CI)**	**IRR (CI)**	**Events/PYS**	**Incidence rate[a] (CI)**	**IRR (CI)**
	All	51/31986.85	1.59 (1.21–2.1)	0.68 (0.51–0.89)	34/19771.38	1.72 (1.23–2.41)	0.73 (0.52–1.02)
	Male	25/19583.94	1.28 (0.86–1.89)	0.62 (0.42–0.91)	20/13013.88	1.54 (0.99–2.38)	0.74 (0.48–1.15)
	Female	26/12402.9	2.1 (1.43–3.08)	0.73 (0.5–1.08)	14/6757.5	2.07 (1.23–3.5)	0.72 (0.43–1.22)
	Age group						
	0–4 years	23/21336.56	1.08 (0.72–1.62)	0.43 (0.28–0.64)	7/6556.98	1.07 (0.51–2.24)	0.42 (0.2–0.89)
	5–9 years	12/4122.91	2.91 (1.65–5.13)	1.23 (0.7–2.17)	9/5006.7	1.8 (0.94–3.45)	0.76 (0.4–1.46)
	10–14 years	9/2672.16	3.37 (1.75–6.47)	1.64 (0.86–3.16)	15/6734.93	2.23 (1.34–3.69)	1.09 (0.66–1.8)
	15–19 years	7/3855.21	1.82 (0.87–3.81)	0.88 (0.42–1.84)	3/1472.78	2.04 (0.66–6.32)	0.98 (0.32–3.05)

CI, 95% confidence interval; IRR, incidence rate ratio; PYS, person-years. [a] Per 1000 person-years.

Supplementary Table S4. Incidence Rates of Stress/Adjustment Disorder in the Microtia Cohort and the Control Cohort.

Outcome	Age/sex	All microtia with or without surgery			Control cohort		
		Events/PYS	Incidence rate[a] (CI)	IRR (CI)	Events/PYS	Incidence rate[a] (CI)	IRR (CI)
Stress/adjustment disorders	All	49/51758.23	0.95 (0.72–1.25)	0.82 (0.62–1.09)	1197/1043060.9	1.15 (1.08–1.21)	1 (0.94–1.06)
	Male	29/32597.83	0.89 (0.62–1.28)	0.84 (0.58–1.21)	698/660234.36	1.06 (0.98–1.14)	1 (0.93–1.08)
	Female	20/19160.4	1.04 (0.67–1.62)	0.8 (0.52–1.24)	499/382826.54	1.3 (1.19–1.42)	1 (0.92–1.09)
	Age group						
	0–4 years	19/27893.54	0.68 (0.43–1.07)	0.48 (0.31–0.76)	775/551169.75	1.41 (1.31–1.51)	1 (0.93–1.07)
	5–9 years	14/9129.61	1.53 (0.91–2.59)	1.74 (1.03–2.94)	164/186012.81	0.88 (0.76–1.03)	1 (0.86–1.17)
	10–14 years	15/9407.09	1.59 (0.96–2.64)	1.95 (1.18–3.24)	163/199551.1	0.82 (0.7–0.95)	1 (0.86–1.17)
	15–19 years	1/5327.99	0.19 (0.03–1.33)	0.21 (0.03–1.49)	95/106327.25	0.89 (0.73–1.09)	1 (0.82–1.22)
		**Microtia without surgery**			**Microtia with surgery**		
	**Age/sex**	**Events/PYS**	**Incidence rate[a] (CI)**	**IRR (CI)**	**Events/PYS**	**Incidence rate[a] (CI)**	**IRR (CI)**
	All	27/31986.85	0.84 (0.58–1.23)	0.74 (0.5–1.07)	22/19771.38	1.11 (0.73–1.69)	0.97 (0.64–1.47)
	Male	15/19583.94	0.77 (0.46–1.27)	0.72 (0.44–1.2)	14/13013.88	1.08 (0.64–1.82)	1.02 (0.6–1.72)
	Female	12/12402.9	0.97 (0.55–1.7)	0.74 (0.42–1.31)	8/6757.5	1.18 (0.59–2.37)	0.91 (0.45–1.82)
	Age group						
	0–4 years	14/21336.56	0.66 (0.39–1.11)	0.47 (0.28–0.79)	5/6556.98	0.76 (0.32–1.83)	0.54 (0.23–1.3)
	5–9 years	8/4122.91	1.94 (0.97–3.88)	2.2 (1.1–4.4)	6/5006.7	1.2 (0.54–2.67)	1.36 (0.61–3.03)
	10–14 years	5/2672.16	1.87 (0.78–4.5)	2.29 (0.95–5.5)	10/6734.93	1.48 (0.8–2.76)	1.82 (0.98–3.38)
	15–19 years	0/3855.21	N/A	N/A	1/1472.78	0.68 (0.1–4.82)	0.76 (0.11–5.39)

CI, 95% confidence interval; IRR, incidence rate ratio; PYS, person-years; N/A, not applicable. [a] Per 1000 person-years.

Supplementary Table S5. Incidence Rates of Conduct Disorder in the Microtia Cohort and the Control Cohort.

		All microtia with or without surgery			Control cohort		
**Outcome**	**Age/sex**	**Events/PYS**	**Incidence rate[a] (CI)**	**IRR (CI)**	**Events/PYS**	**Incidence rate[a] (CI)**	**IRR (CI)**
Conduct disorders	All	10/51758.23	0.19 (0.1–0.36)	1.82 (0.98–3.37)	111/1043060.9	0.11 (0.09–0.13)	1 (0.83–1.2)
	Male	8/32597.83	0.25 (0.12–0.49)	1.98 (0.99–3.95)	82/660234.36	0.12 (0.1–0.15)	1 (0.81–1.24)
	Female	2/19160.4	0.1 (0.03–0.42)	1.38 (0.34–5.51)	29/382826.54	0.08 (0.05–0.11)	1 (0.69–1.44)
	Age group						
	0–4 years	5/27893.54	0.18 (0.07–0.43)	0.99 (0.41–2.37)	100/551169.75	0.18 (0.15–0.22)	1 (0.82–1.22)
	5–9 years	1/9129.61	0.11 (0.02–0.78)	2.91 (0.41–20.66)	7/186012.81	0.04 (0.02–0.08)	1 (0.48–2.1)
	10–14 years	4/9407.09	0.43 (0.16–1.13)	21.21 (7.96–56.52)	4/199551.1	0.02 (0.01–0.05)	1 (0.38–2.66)
	15–19 years	0/5327.99	N/A	N/A	0/106327.25	N/A	N/A
		**Microtia without surgery**			**Microtia with surgery**		
	**Age/sex**	**Events/PYS**	**Incidence rate[a] (CI)**	**IRR (CI)**	**Events/PYS**	**Incidence rate[a] (CI)**	**IRR (CI)**
	All	6/31986.85	0.19 (0.08–0.42)	1.76 (0.79–3.92)	4/19771.38	0.2 (0.08–0.54)	1.9 (0.71–5.07)
	Male	5/19583.94	0.26 (0.11–0.61)	2.06 (0.86–4.94)	3/13013.88	0.23 (0.07–0.71)	1.86 (0.6–5.75)
	Female	1/12402.9	0.08 (0.01–0.57)	1.06 (0.15–7.56)	1/6757.5	0.15 (0.02–1.05)	1.95 (0.28–13.87)
	Age group						
	0–4 years	3/21336.56	0.14 (0.05–0.44)	0.77 (0.25–2.4)	2/6556.98	0.31 (0.08–1.22)	1.68 (0.42–6.72)
	5–9 years	1/4122.91	0.24 (0.03–1.72)	6.45 (0.91–45.76)	0/5006.7	N/A	N/A
	10–14 years	2/2672.16	0.75 (0.19–2.99)	37.34 (9.34–149.3)	2/6734.93	0.3 (0.07–1.19)	14.82 (3.71–59.24)
	15–19 years	0/3855.21	N/A	N/A	0/1472.78	N/A	N/A

CI, 95% confidence interval; IRR, incidence rate ratio; PYS, person-years; N/A, not applicable. [a] Per 1000 person-years.

Supplementary Table S6. Incidence Rates of Hyperkinetic Disorder in the Microtia Cohort and the Control Cohort.

		All microtia with or without surgery			Control cohort		
**Outcome**	**Age/sex**	**Events/PYS**	**Incidence rate[a] (CI)**	**IRR (CI)**	**Events/PYS**	**Incidence rate[a] (CI)**	**IRR (CI)**
Hyperkinetic disorders	All	152/51758.23	2.94 (2.51–3.44)	5.35 (4.56–6.27)	573/1043060.9	0.55 (0.51–0.6)	1 (0.92–1.09)
	Male	126/32597.83	3.87 (3.25–4.6)	6.05 (5.08–7.2)	422/660234.36	0.64 (0.58–0.7)	1 (0.91–1.1)
	Female	26/19160.4	1.36 (0.92–1.99)	3.44 (2.34–5.05)	151/382826.54	0.39 (0.34–0.46)	1 (0.85–1.17)
	Age group						
	0–4 years	105/27893.54	3.76 (3.11–4.56)	4.01 (3.31–4.85)	518/551169.75	0.94 (0.86–1.02)	1 (0.92–1.09)
	5–9 years	30/9129.61	3.29 (2.3–4.7)	17.98 (12.57–25.71)	34/186012.81	0.18 (0.13–0.26)	1 (0.71–1.4)
	10–14 years	16/9407.09	1.7 (1.04–2.78)	28.28 (17.33–46.17)	12/199551.1	0.06 (0.03–0.11)	1 (0.57–1.76)
	15–19 years	1/5327.99	0.19 (0.03–1.33)	2.22 (0.31–15.74)	9/106327.25	0.08 (0.04–0.16)	1 (0.52–1.92)
		**Microtia without surgery**			**Microtia with surgery**		
	**Age/sex**	**Events/PYS**	**Incidence rate[a] (CI)**	**IRR (CI)**	**Events/PYS**	**Incidence rate[a] (CI)**	**IRR (CI)**
	All	108/31986.85	3.38 (2.8–4.08)	6.15 (5.09–7.42)	44/19771.38	2.23 (1.66–2.99)	4.05 (3.01–5.44)
	Male	85/19583.94	4.34 (3.51–5.37)	6.79 (5.49–8.4)	41/13013.88	3.15 (2.32–4.28)	4.93 (3.63–6.69)
	Female	23/12402.9	1.85 (1.23–2.79)	4.7 (3.12–7.07)	3/6757.5	0.44 (0.14–1.38)	1.13 (0.36–3.49)
	Age group						
	0–4 years	80/21336.56	3.75 (3.01–4.67)	3.99 (3.2–4.97)	25/6556.98	3.81 (2.58–5.64)	4.06 (2.74–6)
	5–9 years	18/4122.91	4.37 (2.75–6.93)	23.89 (15.05–37.91)	12/5006.7	2.4 (1.36–4.22)	13.11 (7.45–23.09)
	10–14 years	9/2672.16	3.37 (1.75–6.47)	56.01 (29.14–107.64)	7/6734.93	1.04 (0.5–2.18)	17.28 (8.24–36.25)
	15–19 years	1/3855.21	0.26 (0.04–1.84)	3.06 (0.43–21.75)	0/1472.78	N/A	N/A

CI, 95% confidence interval; IRR, incidence rate ratio; PYS, person-years; N/A, not applicable. [a] Per 1000 person-years.

Supplementary Table S7. Incidence Rates of Autism in the Microtia Cohort and the Control Cohort.

		**All microtia with or without surgery**			**Control cohort**		
**Outcome**	**Age/sex**	**Events/PYS**	**Incidence rate[a] (CI)**	**IRR (CI)**	**Events/PYS**	**Incidence rate[a] (CI)**	**IRR (CI)**
Autism	All	12/51758.23	0.23 (0.13–0.41)	9.67 (5.49–17.03)	25/1043060.9	0.02 (0.02–0.04)	1 (0.68–1.48)
	Male	11/32597.83	0.34 (0.19–0.61)	12.38 (6.85–22.35)	18/660234.36	0.03 (0.02–0.04)	1 (0.63–1.59)
	Female	1/19160.4	0.05 (0.01–0.37)	2.85 (0.4–20.26)	7/382826.54	0.02 (0.01–0.04)	1 (0.48–2.1)
	Age group						
	0–4 years	12/27893.54	0.43 (0.24–0.76)	15.81 (8.98–27.84)	15/551169.75	0.03 (0.02–0.05)	1 (0.6–1.66)
	5–9 years	0/9129.61	N/A	N/A	6/186012.81	0.03 (0.01–0.07)	1 (0.45–2.23)
	10–14 years	0/9407.09	N/A	N/A	3/199551.1	0.02 (0–0.05)	1 (0.32–3.1)
	15–19 years	0/5327.99	N/A	N/A	1/106327.25	0.01 (0–0.07)	1 (0.14–7.1)
		**Microtia without surgery**			**Microtia with surgery**		
	**Age/sex**	**Events/PYS**	**Incidence rate[a] (CI)**	**IRR (CI)**	**Events/PYS**	**Incidence rate[a] (CI)**	**IRR (CI)**
	All	12/31986.85	0.38 (0.21–0.66)	15.65 (8.89–27.56)	0/19771.38	N/A	N/A
	Male	11/19583.94	0.56 (0.31–1.01)	20.6 (11.41–37.2)	0/13013.88	N/A	N/A
	Female	1/12402.9	0.08 (0.01–0.57)	4.41 (0.62–31.3)	0/6757.5	N/A	N/A
	Age group						
	0–4 years	12/21336.56	0.56 (0.32–0.99)	20.67 (11.74–36.39)	0/6556.98	N/A	N/A
	5–9 years	0/4122.91	N/A	N/A	0/5006.7	N/A	N/A
	10–14 years	0/2672.16	N/A	N/A	0/6734.93	N/A	N/A
	15–19 years	0/3855.21	N/A	N/A	0/1472.78	N/A	N/A

CI, 95% confidence interval; IRR, incidence rate ratio; PYS, person-years; N/A, not applicable. [a] Per 1000 person-years.

Supplementary Table S8. Incidence Rates of Asperger syndrome in the Microtia Cohort and the Control Cohort.

Outcome	Age/sex	All microtia with or without surgery			Control cohort		
		Events/PYS	Incidence rate[a] (CI)	IRR (CI)	Events/PYS	Incidence rate[a] (CI)	IRR (CI)
Asperger syndrome	All	3/51758.23	0.06 (0.02–0.18)	2.88 (0.93–8.93)	21/1043060.9	0.02 (0.01–0.03)	1 (0.65–1.53)
	Male	1/32597.83	0.03 (0–0.22)	1.19 (0.17–8.46)	17/660234.36	0.03 (0.02–0.04)	1 (0.62–1.61)
	Female	2/19160.4	0.1 (0.03–0.42)	9.99 (2.5–39.94)	4/382826.54	0.01 (0–0.03)	1 (0.38–2.66)
	Age group						
	0–4 years	3/27893.54	0.11 (0.03–0.33)	3.29 (1.06–10.21)	18/551169.75	0.03 (0.02–0.05)	1 (0.63–1.59)
	5–9 years	0/9129.61	N/A	N/A	1/186012.81	0.01 (0–0.04)	1 (0.14–7.1)
	10–14 years	0/9407.09	N/A	N/A	2/199551.1	0.01 (0–0.04)	1 (0.25–4)
	15–19 years	0/5327.99	N/A	N/A	0/106327.25	N/A	N/A
		**Microtia without surgery**			**Microtia with surgery**		
	**Age/sex**	**Events/PYS**	**Incidence rate[a] (CI)**	**IRR (CI)**	**Events/PYS**	**Incidence rate[a] (CI)**	**IRR (CI)**
	All	1/31986.85	0.03 (0–0.22)	1.55 (0.22–11.02)	2/19771.38	0.1 (0.03–0.4)	5.02 (1.26–20.09)
	Male	1/19583.94	0.05 (0.01–0.36)	1.98 (0.28–14.08)	0/13013.88	N/A	N/A
	Female	0/12402.9	N/A	N/A	2/6757.5	0.3 (0.07–1.18)	28.33 (7.08–113.26)
	Age group						
	0–4 years	1/21336.56	0.05 (0.01–0.33)	1.44 (0.2–10.19)	2/6556.98	0.31 (0.08–1.22)	9.34 (2.34–37.34)
	5–9 years	0/4122.91	N/A	N/A	0/5006.7	N/A	N/A
	10–14 years	0/2672.16	N/A	N/A	0/6734.93	N/A	N/A
	15–19 years	0/3855.21	N/A	N/A	0/1472.78	N/A	N/A

CI, 95% confidence interval; IRR, incidence rate ratio; PYS, person-years; N/A, not applicable. [a] Per 1000 person-years.

Supplementary Table S9. Incidence Rates of Dysthymia in the Microtia Cohort and the Control Cohort.

		All microtia with or without surgery			Control cohort		
**Outcome**	**Age/sex**	**Events/PYS**	**Incidence rate (CI)**	**IRR (CI)**	**Events/PYS**	**Incidence rate (CI)**	**IRR (CI)**
Dysthymia	All	6/51758.23	0.12 (0.05–0.26)	0.71 (0.32–1.57)	171/1043060.9	0.16 (0.14–0.19)	1 (0.86–1.16)
	Male	1/32597.83	0.03 (0–0.22)	0.22 (0.03–1.58)	91/660234.36	0.14 (0.11–0.17)	1 (0.81–1.23)
	Female	5/19160.4	0.26 (0.11–0.63)	1.25 (0.52–3)	80/382826.54	0.21 (0.17–0.26)	1 (0.8–1.24)
	Age group						
	0–4 years	0/27893.54	N/A	N/A	102/551169.75	0.19 (0.15–0.22)	1 (0.82–1.21)
	5–9 years	4/9129.61	0.44 (0.16–1.17)	2.33 (0.87–6.2)	35/186012.81	0.19 (0.14–0.26)	1 (0.72–1.39)
	10–14 years	1/9407.09	0.11 (0.01–0.75)	0.92 (0.13–6.55)	23/199551.1	0.12 (0.08–0.17)	1 (0.66–1.5)
	15–19 years	1/5327.99	0.19 (0.03–1.33)	1.81 (0.26–12.88)	11/106327.25	0.1 (0.06–0.19)	1 (0.55–1.81)
		**Microtia without surgery**			**Microtia with surgery**		
	**Age/sex**	**Events/PYS**	**Incidence rate (CI)**	**IRR (CI)**	**Events/PYS**	**Incidence rate (CI)**	**IRR (CI)**
	All	3/31986.85	0.09 (0.03–0.29)	0.57 (0.18–1.77)	3/19771.38	0.15 (0.05–0.47)	0.93 (0.3–2.87)
	Male	0/19583.94	N/A	N/A	1/13013.88	0.08 (0.01–0.55)	0.56 (0.08–3.96)
	Female	3/12402.9	0.24 (0.08–0.75)	1.16 (0.37–3.59)	2/6757.5	0.3 (0.07–1.18)	1.42 (0.35–5.66)
	Age group						
	0–4 years	0/21336.56	N/A	N/A	0/6556.98	N/A	N/A
	5–9 years	3/4122.91	0.73 (0.23–2.26)	3.87 (1.25–11.99)	1/5006.7	0.2 (0.03–1.42)	1.06 (0.15–7.54)
	10–14 years	0/2672.16	N/A	N/A	1/6734.93	0.15 (0.02–1.05)	1.29 (0.18–9.15)
	15–19 years	0/3855.21	N/A	N/A	1/1472.78	0.68 (0.1–4.82)	6.56 (0.92–46.59)

CI, 95% confidence interval; IRR, incidence rate ratio; PYS, person-years; N/A, not applicable. [a] Per 1000 person-years.
